# Effects of Sensorineural Hearing Loss on Cortical Synchronization to Competing Speech during Selective Attention

**DOI:** 10.1523/JNEUROSCI.1936-19.2020

**Published:** 2020-03-18

**Authors:** Søren A. Fuglsang, Jonatan Märcher-Rørsted, Torsten Dau, Jens Hjortkjær

**Affiliations:** ^1^Hearing Systems Section, Department of Health Technology, Technical University of Denmark, DK-2800 Kgs. Lyngby, Denmark, and; ^2^Danish Research Centre for Magnetic Resonance, Centre for Functional and Diagnostic Imaging and Research, Copenhagen University Hospital Hvidovre, DK-2650 Hvidovre, Denmark

**Keywords:** decoding, EEG, hearing loss, selective attention, speech, stimulus-response models

## Abstract

When selectively attending to a speech stream in multi-talker scenarios, low-frequency cortical activity is known to synchronize selectively to fluctuations in the attended speech signal. Older listeners with age-related sensorineural hearing loss (presbycusis) often struggle to understand speech in such situations, even when wearing a hearing aid. Yet, it is unclear whether a peripheral hearing loss degrades the attentional modulation of cortical speech tracking. Here, we used psychoacoustics and electroencephalography (EEG) in male and female human listeners to examine potential effects of hearing loss on EEG correlates of speech envelope synchronization in cortex. Behaviorally, older hearing-impaired (HI) listeners showed degraded speech-in-noise recognition and reduced temporal acuity compared with age-matched normal-hearing (NH) controls. During EEG recordings, we used a selective attention task with two spatially separated simultaneous speech streams where NH and HI listeners both showed high speech recognition performance. Low-frequency (<10 Hz) envelope-entrained EEG responses were enhanced in the HI listeners, both for the attended speech, but also for tone sequences modulated at slow rates (4 Hz) during passive listening. Compared with the attended speech, responses to the ignored stream were found to be reduced in both HI and NH listeners, allowing for the attended target to be classified from single-trial EEG data with similar high accuracy in the two groups. However, despite robust attention-modulated speech entrainment, the HI listeners rated the competing speech task to be more difficult. These results suggest that speech-in-noise problems experienced by older HI listeners are not necessarily associated with degraded attentional selection.

**SIGNIFICANCE STATEMENT** People with age-related sensorineural hearing loss often struggle to follow speech in the presence of competing talkers. It is currently unclear whether hearing impairment may impair the ability to use selective attention to suppress distracting speech in situations when the distractor is well segregated from the target. Here, we report amplified envelope-entrained cortical EEG responses to attended speech and to simple tones modulated at speech rates (4 Hz) in listeners with age-related hearing loss. Critically, despite increased self-reported listening difficulties, cortical synchronization to speech mixtures was robustly modulated by selective attention in listeners with hearing loss. This allowed the attended talker to be classified from single-trial EEG responses with high accuracy in both older hearing-impaired listeners and age-matched normal-hearing controls.

## Introduction

One of the most deleterious symptoms of age-related sensorineural hearing loss (presbycusis) is a reduced ability to understand speech in everyday noisy situations. This problem is not always mitigated by amplification of the sounds arriving at the ears, as seen by the fact that many hearing aid users continue to experience substantial difficulties understanding speech in the presence of other sound sources (Kochkin, 2005). Although sensorineural presbycusis is characterized by a degeneration in the cochlea, a range of effects in central auditory processing are likely to influence speech understanding in noisy situations (Peelle and Wingfield, 2016). In young normal-hearing listeners, ongoing low-frequency activity (<10 Hz) in auditory cortex is known to synchronize to slow fluctuations in speech stimuli (Luo and Poeppel, 2007; Aiken and Picton, 2008; Ding and Simon, 2012a; Di Liberto et al., 2015). Selectively listening to one speech stream in a speech mixture has been shown to result in an enhanced cortical representation of the attended speech stream compared with the ignored speech (Ding and Simon, 2012b; Zion Golumbic et al., 2013; O'Sullivan et al., 2015). Yet, it is unclear whether presbycusis may interfere with this attentional modulation of cortical synchronization to competing speech signals.

In situations with competing talkers, speech perception involves at least two distinct processes that could each be hampered by presbycusis (Shinn-Cunningham and Best, 2008). First, the ability to perceptually segregate the individual sound streams from a sound mixture relies on the encoding of spectrotemporal cues that is often degraded in the impaired system (Grimault et al., 2001). Second, successful speech comprehension additionally involves the ability to select relevant speech sources using top-down attention. A failure to segregate speech streams also impairs the ability to attend selectively to particular ones (Dai et al., 2018). Yet, the ability to direct attention to selectively listen to one stream and ignore others may be reduced even in acoustic situations where the competing streams can be segregated.

Normal aging by itself can lead to declines in both auditory processing and selective attention (Van Gerven and Guerreiro, 2016). Previous studies have reported abnormally enhanced responses to sound envelope fluctuations in the central auditory system with progressing age (Walton et al., 2002; Goossens et al., 2016; Presacco et al., 2016a, 2019; Parthasarathy et al., 2019). Although such cortical hyperactivity occurs in older listeners with clinically normal audiometric thresholds (Presacco et al., 2016a), enhanced envelope responses in cortex also occur with peripheral hearing loss (Millman et al., 2017; Goossens et al., 2018). Enhanced envelope representations may help the detection of sounds in a quiet background but may also degrade the perception of simultaneously fluctuating signals (Moore and Glasberg, 1993; Moore et al., 1995). However, aging is also thought to reduce cortical inhibitory control functions that support the ability to suppress interference from task-irrelevant sensory information (Gazzaley et al., 2005, 2008). Older individuals may thus become more easily distracted by irrelevant information regardless of their hearing status (Wingfield and Tun, 2001; Andrés et al., 2006). Presacco et al. (2016b) reported that envelope-entrained responses in older listeners were affected by distracting information to a higher degree than in young listeners. Petersen et al. (2017) reported an enhanced tracking of distractor speech in listeners with presbycusis, but in that study age was correlated with the degree of hearing loss. It is thus unclear to what extent problems with speech understanding in older listeners relate to peripheral deficits and/or to an age-related decline in central attention-related processes.

To dissociate effects of sensorineural hearing loss from age, the present study compared cortical responses to competing speech streams in older listeners with presbycusis and age-matched normal-hearing controls. We used spatially separated speech stimuli presented at sound levels where speech comprehension remains high, but where speech-listening typically is experienced as more effortful for listeners with hearing loss. We asked whether hearing loss in such situations affects the attention-dependent selective cortical synchronization to attended and ignored speech streams.

## Materials and Methods

### 

#### Participants and audiometry

Forty-five subjects participated in this study. It was not possible to obtain scalp electroencephalography (EEG) data from one subject (normal hearing male, 58 years old), who was therefore excluded from the analysis. Hearing-impaired (HI; *N* = 22, 9 females, 19 right handed) and normal-hearing (NH; *N* = 22, 16 females, 18 right handed) subjects between 51 and 76 years of age participated. The HI and NH groups were matched in age (*t*_(41.98)_ = −1.62, *p* = 0.1122; NH: mean age 63.0 ± 7.1; HI: mean age 66.4 ± 7.0). HI listeners were selected to have a steeply sloping high-frequency hearing loss indicating presbycusis (Bisgaard et al., 2010; see Fig. 2*a*). For NH listeners, the inclusion criterion was audiometric thresholds within 20 dB of normal hearing level (HL) at frequencies up to 2 kHz and within 35 dB HL for frequencies >2 kHz. One NH subject had a dip in the audiogram at 8 kHz that was 40 dB HL on the left ear and 30 dB HL on the right ear. To ensure that subjects with thresholds above the standard clinical threshold of 20 dB HL did not bias our results, we computed the same analyses while excluding NH subjects with thresholds >20 dB HL. This resulted in a subgroup of 10 NH listeners with ages up to 69 years. To form an age-matched HI subgroup we then similarly selected HI subjects with ages up to 69 years (resulting in a subgroup of 11 HI subjects). This subgroup analysis with a stricter NH criterion produced qualitatively equivalent results in the EEG response data, and the results for the entire group are reported in the following unless stated otherwise. The absolute difference in pure-tone average (measured at 500, 1000, 2000, and 4000 Hz) between ears was ≤15 dB HL for all subjects. Differences between pure-tone audiometric thresholds across ears were at most 25 dB at individual audiometric frequencies. Bone-conduction thresholds were measured at 0.5, 1, and 2 kHz. All subjects had air-bone gaps less than or equal to 10 dB at any audiometric frequency. Tympanometry and otoscopy screening was used to assure normal middle- and outer-ear function.

All subjects provided written informed consent to participate. The experiment was approved by the Science Ethics Committee for the Capital Region of Denmark (protocol H-16036391) and was conducted in accordance with the Declaration of Helsinki.

#### Speech perception in noise

A Danish hearing-in-noise test (DaHINT; Nielsen and Dau, 2009) was used to estimate speech reception thresholds (SRTs). Listeners were presented with spoken sentences in speech-shaped stationary noise at equal hearing level (65 dB HL) and asked to repeat the sentences. The stimuli were presented diotically using Sennheiser HD650 headphones in a double-walled sound booth. The level of the speech signal varied adaptively to identify reception thresholds for each subject, indicating the signal-to-noise ratio (SNR) at which the listeners correctly recognize 50% of the presented sentences. Each listener was presented with 3 different lists consisting of 20 sentences, and the SRTs were averaged across lists.

#### Temporal processing acuity

A psychoacoustic tone-in-noise detection test (adapted from Larsby and Arlinger, 1999) was used to assess temporal processing acuity. A pulsating pure tone (500 Hz, 275 ms duration, 2.22 pulses/s) was presented in different background noise conditions. First, the threshold for tone detection was measured in wide-band noise with a passband corresponding to six equivalent rectangular bandwidths (Moore, 1986) around the target tone frequency. Next, a temporal gap in the noise of 50 ms centered on the tone was introduced. The temporal masking release, i.e., the difference in detection thresholds between the no-gap and gap conditions, was then calculated as a measure of listeners' abilities to use temporal fluctuations in the noise masker for improved detection. The noise was presented at a fixed sound pressure level (SPL) of 55 dB and the level of the target tone was varied using a Békésy tracking procedure to identify the thresholds. The subjects performed each condition (no-gap, temporal gap) twice for each ear. Subjects also performed a spectral gap detection not included in the analysis. The stimuli were presented using Sennheiser HDA200 headphones in a double-walled sound booth.

#### Working memory performance

Speech perception in noise by older listeners may not only depend on their hearing status but also on cognitive abilities (Akeroyd, 2008) and hearing impairment may itself affect cognitive function (Wingfield and Peelle, 2012). To ensure that the recruited older NH and HI listeners were matched in cognitive abilities, a reversed digit span test was used to measure working memory performance. In the test, listeners were asked to recall a presented sequence of numbers (between 1 and 9) in reverse order. The digit span score was then calculated as the number of items that could be repeated correctly (Blackburn and Benton, 1957). The auditory stimuli were presented via Sennheiser HD650 headphones at a comfortable level (70 ± 10 dB SPL). The listeners first performed a forward digit span to familiarize them with the procedure.

#### Self-evaluated hearing disabilities

All subjects completed the Speech, Spatial, and Qualities of Hearing Scale questionnaire (SSQ; Gatehouse and Noble, 2004). The SSQ questionnaire consists of 49 questions related to self-rated hearing abilities in everyday situations. The questions address hearing in three domains: “Speech” (e.g., comprehending speech and selectively attending to a particular talker in everyday listening situations), “Spatial” (e.g., judging direction, distance, and movement of sound sources), and “Qualities” (e.g., segregation of sound sources, clarity, and listening effort).

#### Accounting for reduced audibility

The speech stimuli in the DaHINT and EEG experiments were amplified based on the HI listeners' audiometric thresholds to account for reduced audibility. A linear gain was applied at each audiometric frequency according to the Cambridge formula (CamEQ; Moore and Glasberg, 1998) and was limited to 30 dB gain at a given frequency. The level of the speech stimuli was 65 dB SPL before the frequency-dependent amplification (i.e., equalization). The tone stimuli used in the EEG experiments were presented at a comfortable listening level per subject.

#### EEG experiments

The EEG experiments were performed in an electrically shielded double-walled sound booth. In all EEG experiments, the subjects were comfortably seated and instructed to fixate their eye-gaze at a cross hair presented on a computer screen. EEG data were recorded using a BioSemi Active-Two system with 64-scalp electrodes positioned according to the 10–20 system. Two additional bipolar electrooculography electrodes were mounted above and below the left eye. The EEG data were digitized at a sampling rate of 512 Hz. EEG was also measured inside the ear canals in some subjects, but the ear EEG data were not included in the analysis. The auditory stimuli were presented via ER-3 insert earphones (Etymotic Research). Resting EEG data were also recorded but not considered for analysis.

#### Tone stimuli

Envelope-following responses (EFRs) were recorded from subjects listening passively to tone sequences designed to induce cortical activity in the gamma (40 Hz) and theta (4 Hz) frequency ranges. The stimuli are illustrated in Figure 5*a*. Two types of stimulation paradigms were used. In both, 1 kHz tone pulses (10 ms Hann-shaped ramps) with an interpulse interval of 25 ms were presented in epochs of 2 s stimulation, alternating with 1 s periods of silence. In the first stimulation paradigm, 0.5-s-long 40 Hz tone sequences alternated with 0.5-s-long silence intervals, resulting in a periodic 4 Hz onset/offset pattern (see Fig. 5*a*, top). In the second paradigm, no 4 Hz onset/offset pattern was imposed (see Fig. 5*a*, bottom). In each of the two stimulations, 60 3-s-long epochs were presented.

Event related potentials (ERPs) during passive listening to 1 kHz pure tones were also recorded. The tone stimuli had a duration of 100 ms and were ramped using a 10 ms long Hann window. The tones were presented at an average inter-tone interval rate of 1 s that was randomly jittered ±25 ms. Each subject listened to 180 tone repetitions.

#### Selective speech attention experiment

The main experiment was designed to measure cortical responses to competing speech streams during a selective attention task. EEG data were recorded from subjects selectively listening to one of two simultaneous speech streams or to a single speech stream in quiet. The speech stimuli consisted of two different audiobooks read by a male and a female speaker. Prolonged silent periods in the speech stimuli were truncated to be 450 ms long. The audio files were split into ∼50-s-long trials. The speech streams were spatially separated at ±90° using non-individualized head related transfer functions (HRTFs) provided by Oreinos and Buchholz (2013). The audio files were low-pass filtered at 12 kHz using a second-order Butterworth filter to avoid excessive high-frequency amplification for subjects with low audiometric thresholds. The audio signals of the two talkers were matched in loudness before spatialization according to ITU standard ITU-R BS.1770-1. Loudness matching was used to obtain EEG responses to the two speech streams that were not influenced by systematic differences in sound level between target and masker speech. Subjects were asked to judge the perceived loudness of the two speech streams after the experiment, and all reported that the loudness was perceived to be similar in level.

**Figure 1.**
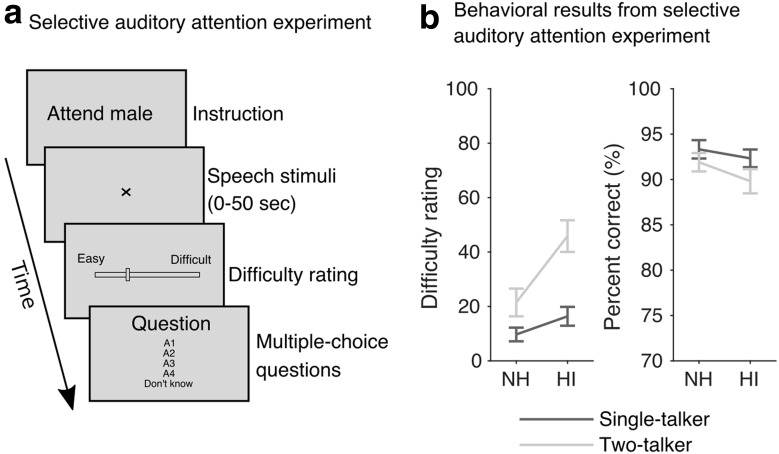
Auditory attention EEG experiment. ***a***, Schematic illustration of the trial sequences. EEG data were recorded from subjects selectively listening to audiobooks narrated by a male and a female speaker. After each trial, the subjects were asked to rate task difficulty and respond to multiple-choice questions related to the content of the attended speech stream. ***b***, Behavioral results from the selective auditory attention experiment showing difficulty rating scores (left) comprehension scores (right). Error bars indicate SEM.

Figure 1*a* presents the trial structure of the selective listening experiment. In ∼50-s-long trials, the subjects listened to either a single talker or two competing talkers. The experiment consisted of 48 trials. Each subject listened to 2 blocks of 12 trials with the male speaker as the target, and 2 blocks of 12 trials with the female speaker as the target. Each block of 12 trials consisted of 4 single-talker trials, and 8 two-talker trials. At the onset of each trial, the subject was instructed to attend to either the male or the female talker. As an additional cue, the target speech stream was switched on ∼4 s (jittered between 3 and 5 s) before the interfering speech stream. The EEG data recorded in this period were discarded from analysis. The number of left versus right target trials was balanced across the experiment. After each trial, the subjects were asked to rate how easy or difficult it was to understand the attended speech in that trial on a continuous rating scale marked 'easy' and 'difficult' at the extremes. On average, subjects reported that it was more difficult to follow the male speaker compared with the female speaker. However, the number of trials in which the subjects attended to the male and to the female speaker was balanced within subject. After the rating, listeners were prompted to answer four multiple-choice comprehension questions related to the content of the attended speech stream. The first of the four comprehension questions was also shown before the trial started. Subjects were given feedback on their responses.

#### Data analysis

##### EEG preprocessing.

EEG data analyses were performed with MATLAB (R2018b, MathWorks) using the Fieldtrip toolbox (20190207; Oostenveld et al., 2011) and the Gramm toolbox for figures (Morel, 2016). The digitized EEG data were re-referenced to the average of electrodes TP7 and TP8. EEG data recorded during the selective attention experiment and the EEG data used for extraction of ERPs were low-pass filtered at 30 Hz using a windowed 226th-order linear phase finite impulse response (FIR) filter. The EFR data were low-pass filtered at 60 Hz using a 114th-order linear phase FIR filter. Line noise (50 Hz) was removed via notch filtering for the ERP and EFR data. The data were then downsampled to 128 Hz for EFR and ERP data and to 64 Hz for the EEG recorded during the selective attention experiment. The ERP and EFR data were subsequently high-pass filtered at 0.1 Hz (VanRullen, 2011; Rousselet, 2012), using a 2112th-order linear phase FIR filter. The EEG data from the attention experiment were high-pass filtered at 0.5 Hz using a 212th-order linear phase FIR filter. The data were segmented into epochs and electro-ocular (EOG) artifacts were removed (see next paragraph for details). The downsampled and de-noised EEG data from the selective attention experiment were finally filtered between 1 and 9 Hz. This was done by first applying a 106th-order linear phase FIR high pass filter with a 1 Hz cutoff and then low-pass filtering the data with a 94th-order linear phase FIR filter with a 9 Hz cutoff. The data were in all cases shifted to account for the filter delays.

A joint decorrelation framework (de Cheveigné and Parra, 2014) was used to remove EOG artifacts from the EEG speech and ERP data similarly as described by Wong et al. (2018). The mean of each electrode response was first stored and subtracted from the data. Data segments containing EOG artifacts were detected using the Hilbert envelopes of EOG channel responses and responses over three frontal electrodes, Fp1, Fpz, and Fp2 and two additional EOG electrodes. The Hilbert envelope of each of these channel responses was extracted after bandpass filtering (passband: 2–15 Hz, 4th-order Butterworth filter), then *z*-scored and collapsed into one channel. Time points where the resulting signals exceeded a threshold of four were considered artifactual. The artifactual segments were extended by 0.1 s on both sides (as implemented in Fieldtrip; Oostenveld et al., 2011). The labeled segments were then used to compute an artifact biased covariance matrix. The estimated artifact biased covariance matrix and the covariance matrix estimated from the entire dataset were whitened via principal component analysis. Eigenvectors characterizing the maximum variance differences between the two covariance matrices were then computed (de Cheveigné and Parra, 2014; Wong et al., 2018) using the NoiseTools toolbox (http://www.audition.ens.fr/adc/NoiseTools). This defines a spatial filter that was then used to regress out EOG artifacts. Eigenvectors with eigenvalues >80% of the maximum eigenvalue were subsequently regressed out from the data (Wong et al., 2018). The mean electrode response that had been subtracted before de-noising was added to the de-noised data.

##### Extracting ERPs.

For the tone response data (ERP/EFR), the mean amplitude of the N1 component (van Diepen and Mazaheri, 2018) was examined in the time window from 75 to 130 ms post-onset. For this analysis, the EFR data were preprocessed in the same way as for the ERP data. The data were averaged over a subset of 14 fronto-central electrodes (FC5, FC3, FC1, FCz, Fz, FC2, FC4, FC6, F5, F3, F1, F2, F4, F6).

##### EFR intertrial phase coherence.

The inter trial phase coherence (ITPC) was computed for EEG responses to EFR stimuli. To this end, a time-frequency decomposition of each electrode response was performed by convolving the EEG responses with complex Morlet wavelets with a fixed number of 12 cycles per wavelet, as implemented in Fieldtrip. No spatial filtering was performed before the analysis. With *f*_0_ representing the passband center frequency of each Morlet wavelet, we considered an *f*_0_ range between 1 and 50 Hz with a resolution of 0.5 Hz and a temporal resolution of 2/128 s for visualization purposes. The complex output, *F_k_*(*f*,*t*,*n*), for trial *k* = 1, …, *N*, electrode *n*, time bin *t* and center frequency *f* were then used to compute the ITPC:

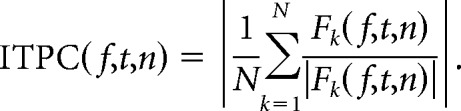
 The ITPC ranges between 0 and 1, and indicates the degree of phase consistency of EEG responses to the EFR stimuli over trials (0 corresponds to no consistency and 1 indicates full consistency). The ITPC was calculated in non-overlapping windows of 0.5 s to investigate potential changes over the 2 s stimulation period. For the statistical analysis of the EFR data, the average of all scalp electrodes was considered.

##### Speech envelope extraction.

The speech envelopes of the attended and unattended speech streams were extracted using a simplistic functional model of the auditory periphery. The monaural versions of the audio stimuli were used, i.e., stimuli that had been collapsed via averaging across channel after spatialization. The audio waveforms (digitized at 44.1 kHz) were low-pass filtered at 6000 Hz using a 98th-order linear-phase FIR filter, downsampled to 12,000 Hz and passed through a “gammatone” filter bank consisting of 24 fourth-order gammatone bandpass filters with center frequencies on an equivalent rectangular bandwidth scale (ranging between 100 and 4000 Hz; Glasberg and Moore, 1990) and 0 dB attenuation at their individual center frequencies. This was based on the implementation available in the Auditory Modeling Toolbox (Søndergaard and Majdak, 2013). The output from each gammatone filter was full-wave rectified and power-law compressed, |*x*|*^c^*, with *c* = 0.3 to mimic the compressive response of the inner ear. The output sub-band envelopes were averaged across gammatone frequency channels to obtain a univariate temporal envelope. The envelope was then low-pass filtered at 256 Hz using a 620^th^-order linear phase FIR filter and resampled to 512 Hz. The envelope was then further low-pass filtered at 30 Hz using a 226th-order linear phase FIR filter and resampled to 64 Hz to match the sampling rate of the EEG data. Finally, the envelope was bandpass filtered as the EEG data between 1 and 9 Hz by first applying a 106th-order FIR high pass filter with a 1 Hz cutoff and then low-pass filtering the data with a 94th-order FIR filter with a 9 Hz cutoff. The filtered data were shifted to adjust for filter delays.

##### Encoding and decoding models.

Following a number of previous speech-attention studies (Ding and Simon, 2012b, 2013), we considered two complementary analyses of statistical stimulus-response dependencies between the envelope of the attended and unattended speech streams and the EEG responses. We considered both forward regression models (encoding models), and backward regression models (decoding models). The encoding models attempt to predict neural responses to speech stimuli, *R*(*t,n*), from the time-lagged speech envelopes *S*(*t*):

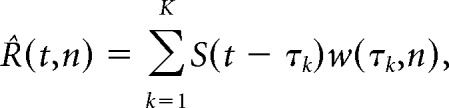
 where *R̂*(*t,n*) is an estimate of the EEG response at a given electrode, *n* = 1,2, … *N*, and *w*(τ*_k_*, *n*) represent the regression weights that define a temporal response function. For the encoding analysis, time lags, τ*_k_* = {τ_1_, τ_2_, …,τ*_K_*}, ranging between 0 and 500 ms poststimulus were considered.

The backward decoding model, on the other hand, integrates information over all EEG electrodes and all time lags to reconstruct the speech envelope:

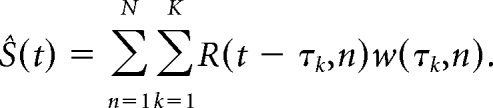
 For the decoding analysis, we considered time-lags τ*_k_* = {τ_1_, τ_2_, …,τ*_K_*}, ranging between −500 and 0 ms poststimulus. For both encoding and decoding models, we included data from 6 s after trial onset (i.e., after the onset of any masking stimulus) to 43 s after trial onset.

The weights of the linear regression models were estimated via ridge regression. Let ***X*** be a standardized matrix and let ***X*** = ***UDV****^T^* be the singular-value decomposition of ***X***. Similarly, let ***Y*** be a vector with zero mean and unit SD. The linear regression model can now be formulated as follows:

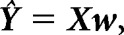
 where ***Ŷ*** is an estimate of ***Y***. The Ridge regression estimator then takes the following form:

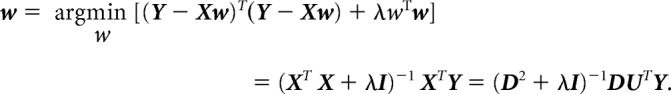
 In the case of a forward encoding model, ***X*** is a matrix containing the speech envelope, *S*(*t*), at multiple time lags and ***Y*** is the EEG response at a given channel. In this case, separate Ridge parameters are estimated for each electrode, each subject and each experimental condition. In the case of a backward model, ***X*** is a matrix containing the multichannel and time-lagged EEG response and ***Y*** is the speech envelope.

To assess the predictive performance of each model we used a nested cross-validation procedure. The nested cross-validation procedure consisted of an outer tenfold cross-validation loop and an inner fivefold cross-validation loop. The data were split 10 times into a training set and a test set, and for each split we further divided the training data randomly into five parts to optimize the Ridge λ parameter. In this way, the Ridge parameter was tuned on the training set and the generalization error was evaluated on the held-out test set. During model fitting and evaluation, the data were standardized to the empirical mean and unit SD of the data used for model fitting. The prediction accuracy was indexed by the Pearson's correlation coefficient between the model prediction ***Ŷ*** and the target data, ***Y***. Pearson's correlation coefficient was chosen as the metric because it ranges between −1 and +1 and is invariant to scaling and shift errors in the predictions. The prediction accuracy was estimated as the average over the 10 initial splits. The performance of the stimulus–response models was in all cases evaluated on data from trials that had not been used for model fitting or parameter tuning.

For the statistical analysis of the results from the encoding analyses, we averaged the encoding accuracies over the same subset of fronto-central electrodes as in the ERP analysis. The noise floor was estimated as by Wong et al. (2018) by phase scrambling target regressors (Prichard and Theiler, 1994). The noise floor was estimated based on aggregated surrogate data from all subjects and all stimulus–response models (i.e., attended single-talker, attended two-talker, and unattended two-talker).

Our stimulus–response analyses were based on envelopes extracted via auditory models that assume a healthy auditory system. Because hearing loss may change this representation, we conducted control analyses to understand whether our results were influenced by these assumptions. First, hearing loss may be associated with a reduced compressive response of the inner ear. To understand whether the results are influenced by the amount of compression assumed by our model, we performed the same analyses with joint encoding models trained on speech envelopes that were compressed with a range of compression factors (c = {0.1,0.2,0.3, …,1}). This analysis yielded equivalent results indicating that the choice of compressive factor (*c* = 0.3) did not introduce a group-level bias in model prediction accuracies. Second, hearing loss may distort the coding of envelope modulations in a frequency-specific way that is not captured by a model assuming a broadband envelope representation. We therefore performed the encoding analyses with models trained on frequency-decomposed cochleograms of the binaural stimuli. We used a gammatone filter bank consisting of 34 filters with center frequencies on an equivalent rectangular bandwidth scale ranging from 100 and to 12,000 Hz, allowing the encoding models to capture modulations in high-frequency critical-bands. This analysis also yielded very similar results, suggesting that the univariate envelope extraction procedure did not introduce biases in the group-level comparisons. Finally, we checked whether the amplification of the audio stimuli would influence the results by performing the stimulus-response analyses both with and without the CamEQ equalization. This also yielded highly similar results. The results from the analyses obtained with the non-equalized stimuli are reported in the following.

The ability to decode attention, i.e., to discriminate between attended and unattended speech envelopes from the EEG data, provides a complimentary measure of how robustly the envelope-entrained responses are modulated by attention. We therefore additionally trained backward models on the single-talker data and then used the models to reconstruct the speech envelopes of the attended talker in the remaining two-talker EEG data. To ensure an unbiased decoding, the Ridge parameter and EOG de-noising filters were fitted based on the single-talker data. For testing, we considered non-overlapping EEG decoding segments of 10 s duration (taking into account the 0.5-s-long kernel of the stimulus reconstruction models) shifted by 15 s long time shifts. To evaluate the attention decoding accuracy, we computed the Pearson's correlation coefficient between the reconstructed envelopes and the envelopes of the attended (*r*_attended_) and unattended (*r*_unattended_) speech streams (O'Sullivan et al., 2015). We considered a classification to be correct whenever the neural reconstruction was more correlated with the envelope of the actual attended speech stream than with the envelope of the unattended speech stream (i.e., *r*_attended_ > *r*_unattended_). Chance-level classification was assumed to follow a binomial distribution.

#### Statistical analysis

Repeated-measures ANOVAs were used to analyze the results from the stimulus–response analyses in the single-talker and the two-talker conditions for attended speech at the group level. Repeated-measures ANOVAs were also used for group-level analysis of the average ITPC results in short time windows. Welch's *t* tests were used to compare psychophysical results (speech-in-noise scores, SSQ ratings, frequency-temporal test scores, and digit span scores) and EEG stimulus–response results between the NH and HI listener groups. Pearson's correlation coefficients were transformed using the Fisher *Z*-transformation before statistical analyses. Classification scores, speech comprehension scores, ITPC values and difficulty ratings were arcsin transformed before statistical analyses. When appropriate, we used the false discovery rate (Benjamini and Hochberg, 1995) to correct for multiple comparisons. All statistical tests were conducted using R v3.6.0 (2019-04-26).

#### Data and code accessibility

All data are publicly available at http://doi.org/10.5281/zenodo.3618205. The code is available at https://gitlab.com/sfugl/snhl.

## Results

### Behavioral hearing tests

**Figure 2.**
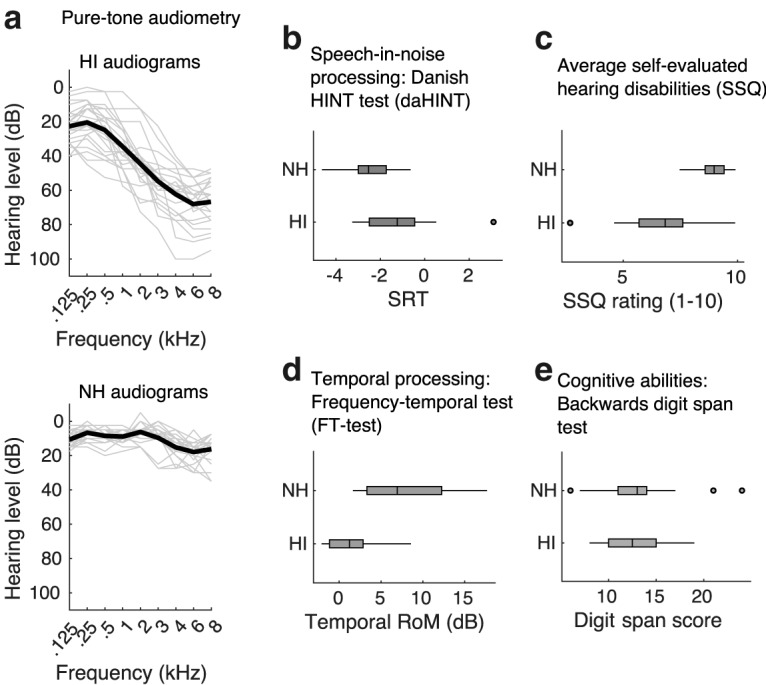
Behavioral hearing tests. ***a***, Pure-tone audiograms for the NH (top) and HI (bottom) subjects. Each thin line represents the audiogram for a single subject averaged over both ears. The thick lines represent averages across subjects. ***b***, SRTs in the two groups measured in a speech-in-noise recognition task. ***c***, Self-assed hearing disabilities as assessed by the SSQ questionnaire. Lower ratings indicate greater self-rated listening difficulties in everyday acoustic environments. The SSQ scores shown here are averaged over three SSQ subsections (speech, qualities, and spatial). ***d***, Tone detection in noise with or without a 50 ms temporal gap. HI listeners showed less temporal release of masking (RoM), i.e., they showed a smaller benefit from temporal gaps in the noise masker compared with NH listeners. ***e***, Working memory performance as measured by a backward digit span test.

Figure 2 summarizes the data of the behavioral hearing tests. For speech-in-noise perception (Fig. 2*b*), HI listeners showed significantly higher sentence reception thresholds compared with the age-matched NH controls (DaHINT test: *t*_(35.78)_ = −3.49, *p* = 0.0013). This reduced speech-in-noise performance was observed despite the fact that the speech stimuli were amplified to account for reduced audibility in the HI listeners. Noticeably, the 50% speech reception thresholds were negative also for most HI listeners and below SNRs typically encountered in everyday environments (Billings and Madsen, 2018).

HI listeners also exhibited a reduced temporal masking release compared with the NH listeners (*t*_(32.74)_ = 5.53, *p* < 0.0001; Fig. 2*d*) suggesting a degraded temporal processing acuity. In the questionnaire data, HI listeners reported greater difficulties with speech listening in everyday listening situations when wearing their own hearing aid. The different SSQ ratings related to spatial hearing, speech perception and sound quality were correlated (Spearman's rank correlations: *r*_(SSQspeech,SSQspatial)_ = 0.79, *r*_(SSQspeech,SSQquality)_ = 0.82, *r*_(SSQspatial,SSQquality)_ = 0.86). The SSQ ratings averaged across the three response categories were significantly lower for the HI group compared with the NH listeners (*t*_(33.10)_ = 6.49, *p* < 0.0001; Fig. 2*c*). Finally, the reversed digit span test confirmed similar working memory performance in the age-matched normal-hearing and hearing-impaired listeners (*t*_(37.85)_ = 0.68, *p* = 0.5016; Fig. 2*e*).

### Behavioral results from selective attention experiment

During the EEG speech listening experiments, listeners responded to speech comprehension questions and rated speech-listening difficulty. These behavioral results are shown in Figure 1*b*. Both normal-hearing and hearing-impaired listeners showed accurate speech comprehension, both in the single-talker condition and in the condition with two competing talkers. A repeated-measures ANOVA showed no significant effect of hearing impairment on speech comprehension scores (*F*_(1,42)_ = 1.31, *p* = 0.2598), but a main effect of talker condition (single vs two talkers; *F*_(1,42)_ = 8.42, *p* = 0.0059). Although the two listener groups answered the comprehension questions with high accuracy, the HI listeners rated the competing speech listening task to be significantly more difficult compared with the NH listeners and compared with the single-talker condition (main effect of hearing impairment on difficulty ratings: *F*_(1,42)_ = 10.5, *p* = 0.0023; Fig. 1*b*, left).

### Speech envelope entrainment during selective attention

**Figure 3.**
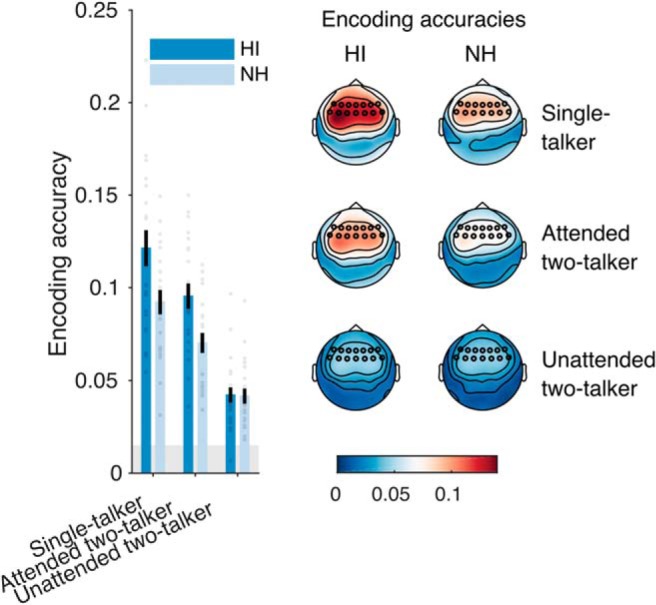
Results from the encoding analysis. **Left**, Group mean encoding accuracies averaged over fronto-central electrodes. Encoding accuracies indicate the correlation between speech envelope model predictions and EEG data from NH (light blue) and HI (dark blue) listeners. Each point represents data from a single subject. Error bars represent SEM. The shaded area indicates the estimated noise floor. **Right**, Topographies showing group-mean encoding accuracies at each electrode site.

In the speech attention experiments, normal-hearing and hearing-impaired subjects listened to speech in quiet or to speech masked by a competing talker. To investigate EEG correlates of cortical speech envelope entrainment in the two groups, we used forward and backward stimulus-response models. Forward model prediction accuracies, i.e., the correlation between the low-frequency EEG response and the response predicted by the envelope model, are shown in Figure 3. A repeated-measures ANOVA was used to test the effect of hearing impairment on the speech envelope entrainment as measured by the EEG prediction accuracies of the forward model on fronto-central electrodes. Envelope entrainment to the target speech was enhanced in HI listeners compared with NH controls in both the single-talker and in the two-talker listening conditions. Main effects of hearing status (NH vs HI; *F*_(1,42)_ = 7.57, *p* = 0.0087) and stimulus condition (single-talker vs two-talker; *F*_(1,42)_ = 54.23, *p* < 0.0001) were found, but no significant interaction between the two (*F*_(1,42)_ = 0.42, *p* = 0.5195). No effect of hearing status on the entrainment for the unattended speech was found (*t*_(42.00)_ = −0.10, *p* = 0.9204).

As a complimentary measure of speech envelope entrainment, the backward model reconstructs the envelope of attended and unattended speech streams from a weighted response of all EEG electrodes. Analysis of the envelope reconstruction accuracies between groups again showed a main effect of hearing status (*F*_(1,42)_ = 13.76, *p* = 0.0006), and stimulus condition (single-talker vs two-talker; *F*_(1,42)_ = 56.03, *p* < 0.0001), indicating again an enhanced envelope representation in the HI listener group. No effect of hearing impairment was observed on the reconstruction accuracies for the unattended speech (*t*_(39.69)_ = −1.34, *p* = 0.1885).

**Figure 4.**
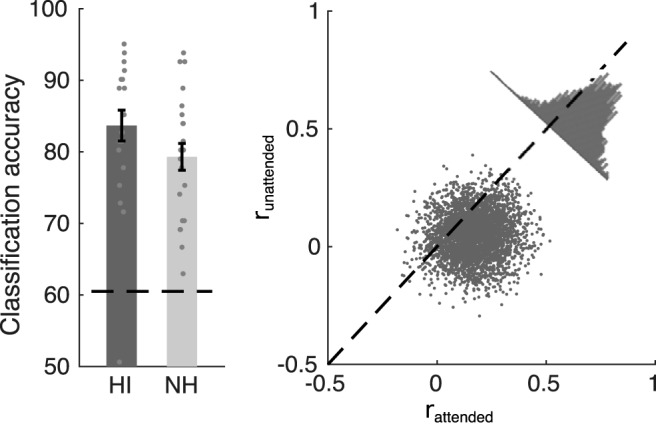
Results of the attention decoding analysis. Stimulus reconstruction models trained on EEG responses to single-talker speech stimuli were used to decode the attended target from 10-s-long EEG responses to two-talker stimuli. For a given test segment, correct classification indicates that the correlation with the speech envelope of the attended stream *r*_attended_ was higher than the unattended *r*_unattended_. **Left**, Attention classification accuracies in NH and HI listeners. Each point represents data averaged from a single subject. The dashed line represents chance-level. Error bars indicate SEM. **Right**, Single-trial reconstruction accuracies for each 10-s-long decoding segment. Each point reflects accuracies for a given subject and a given decoding segment. Data are here shown for all subjects and all 10 s decoding segments.

Both analyses suggested a robust differential entrainment to the attended and unattended speech signals in both groups, similar to what has been previously reported for NH listeners (Ding and Simon, 2012a; O'Sullivan et al., 2015). We next investigated the degree to which this differential response could be used to decode the attentional focus (the attended talker) from single-trial EEG responses. Here, we used the backward models trained on data from single-talker trials. The models were then used to identify the attended target in the EEG responses to the two-talker mixtures. Accurate attention classification here does not by itself necessarily suggest that *r*_attended_ is high, but only that it is higher than *r*_unattended_. Figure 4 shows the results of the attention decoding analysis. We found that the reconstructed envelopes reliably discriminated between attended and unattended speech in both groups of listeners. The mean classification accuracy for 10 s long EEG segments was 83.7% for the HI listeners and 79.3% for the NH listeners and we found no effect of hearing loss on the attention classification accuracies (*t*_(41.40)_ = −1.64, *p* = 0.1077). Restricting the audiometric criterion for normal hearing (<20 dB HL; see Materials and Methods) yielded a significant effect of hearing loss on classification accuracy (*t*_(16.91)_ = −3.074, *p* = 0.0069).

The lack of an effect of hearing loss on attention classification in the main analysis may seem puzzling given the enhanced envelope responses for attended speech in the HI listeners. For attention classification, however, we used models trained on single-talker data to predict the envelope of both the target and the nontarget speech. This was motivated by the fact that a classification system, e.g., in an EEG-controlled BCI, typically does not know in advance the “ground truth” of which speakers are attended. In the envelope-entrainment analysis (Fig. 3), on the other hand, separate response functions were estimated for attended and unattended speech. The rationale for this was that response functions for attended and unattended speech are different (Ding and Simon, 2012a; O'Sullivan et al., 2015), possibly reflecting distinct neural mechanisms related to attending and suppressing the target and nontarget speech streams, respectively. When examining correlations to the nontarget speech predicted by “attended” models trained on single-talker speech data, we observed an enhanced correlation in the HI listeners, both in the forward (*t*_(41.87)_ = −2.28, *p* = 0.0276) and backward model (*t*_(41.53)_ = −3.27, *p* = 0.0022) correlations. The reason for this is unclear, but could for instance arise if listeners momentarily switch their attention to the nontarget speech, or, if the response functions for attended and unattended speech are correlated, in which case a change in attended response functions in HI listeners could affect the correlations between model predictions and nontarget speech.

### Envelope entrainment to tones during passive stimulation

**Figure 5.**
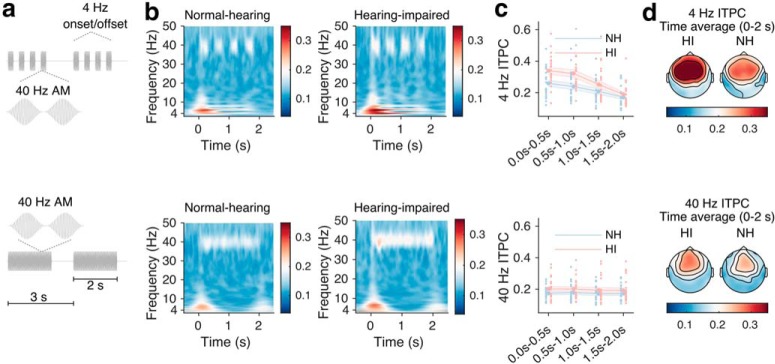
EFR responses to periodic tone sequences during passive stimulation. Top row, EFR stimuli and responses to 0.25 s on/off segments of 40 Hz tone stimuli. Bottom row, Stimuli and responses to 2-s-long 40 Hz tone stimuli. ***a***, EFR tone stimuli. ***b***, Time-frequency representations of ITPC for NH (left) and HI (right) listeners for the two types of stimuli. ***c***, ITPC averaged over all scalp electrodes for NH and HI listeners, in non-overlapping 0.5-s-long time intervals. Shaded areas indicate SEM. The individual red (HI) and blue (NH) points represent data from each subject. ***d***, Topographies of group-mean ITPC at each electrode in the stimulation period (0–2 s).

The speech experiments with single and competing talkers both suggested an enhanced envelope entrainment to attended speech in HI listeners, possibly indicating a stimulus-driven effect. Next, we recorded responses to tone sequences during passive stimulation to obtain measures of stimulus-driven cortical entrainment. We used periodic tone stimuli designed to entrain steady-state cortical activity in the gamma (40 Hz) or theta (4 Hz) range (stimuli illustrated in Fig. 5*a*). Specifically, periodic 4 Hz tone stimulation examines envelope response entrainment in the frequency range also examined in the analysis of the speech stimuli (<10 Hz), but without the attention task component. We computed the ITPC to assess how precisely EEG activity synchronized to the periodic tone stimulation. We computed the ITPC in time windows of 0.5 s to investigate potential differences in ITPC over the 2 s stimulation period. Figure 5*b–d* shows the ITPC results for the two types of tone stimuli. For the 4 Hz stimulation (Fig. 5*c*, top row), a repeated-measures ANOVA on the ITPC revealed a main effect of hearing impairment (*F*_(1,42)_ = 5.00, *p* = 0.0306), a main effect of time (*F*_(3,126)_ = 85.18, *p* < 0.0001) as well as an interaction effect (*F*_(3,126)_ = 5.68, *p* = 0.0011). *Post hoc t* tests revealed that the 4 Hz ITPC was significantly higher (after applied FDR corrections, *q* = 0.05) for HI than for NH in the time period 0–1 s post-onset, but not in the later part of the stimulation (0–0.5 s: *t*_(41.92)_ = −2.82, *p* = 0.0072; 0.5–1.0 s: *t*_(41.44)_ = −2.78, *p* = 0.0081; 1.0–1.5 s: *t*_(41.43)_ = −1.57, *p* = 0.1240; 1.5–2.0 s: *t*_(41.92)_ = −0.93, *p* = 0.3583). On the other hand, the ITPC for 40 Hz stimulation (Fig. 5*c*, bottom row) showed no main effect of hearing loss (*F*_(1,42)_ = 1.65, *p* = 0.2058), or time (*F*_(3,126)_ = 1.36, *p* = 0.2572). As indicated in the topographies (Fig. 5*d*), the ITPC at both rates were prominent at fronto-central electrodes and showed no lateralization effects.

### ERP tone responses

Enhanced envelope-following responses to tones or speech could potentially be driven by an overall enhanced cortical reactivity to sound transients (Aiken and Picton, 2008) following hearing loss. To test whether HI affects transient-evoked EEG responses (Alain et al., 2014), we measured ERP responses to 1 kHz tone beeps presented with random inter-onset intervals during passive listening. No effect of hearing impairment was found on the mean amplitude of the N1 component (*t*_(41.97)_ = 0.83, *p* = 0.4092). We also extracted ERPs elicited by the individual tones in the periodic EFR tone stimuli discussed above. Again, no effect of hearing impairment on the mean N1 amplitudes was observed, neither for the 4 Hz (*t*_(40.07)_ = 0.015, *p* = 0.9883) nor the 40 Hz EFR stimuli (*t*_(41.71)_ = 1.77, *p* = 0.0847). This also indicates that observed changes in phase coherence of the EFR responses with hearing loss are not driven by changes in the amplitude of transient evoked activity (van Diepen and Mazaheri, 2018).

## Discussion

Speech-in-noise listening difficulties are among the most severe consequences of presbycusis, often persisting even when loss of audibility is accounted for, e.g., by a hearing aid (Kochkin, 2005). Here, we investigated effects of sensorineural hearing loss on cortical processing of competing speech. Behaviorally, questionnaire data (SSQ) confirmed that HI listeners, compared with age-matched NH controls, experience significantly greater listening difficulties in everyday noisy situations (while using their hearing aid). This was mirrored by elevated speech reception thresholds in stationary noise (Fig. 2*b*). Although elevated, SRTs for HI listeners remained negative for most subjects. Conversational speech in daily life most often occurs at positive signal-to-noise ratios (Olsen, 1998; Billings and Madsen, 2018) and rarely at noise levels where only half of the speech can be recognized (i.e., 50% SRTs as considered here). In our EEG experiments, we recorded EEG from subjects listening to competing speech signals presented in scenarios with good speech recognition in both listener groups. Yet, listening to speech with a competing talker was rated as being significantly more difficult for listeners with hearing loss compared with NH controls (Fig. 1).

Previous work has associated hearing loss with weakened differential responses to competing streams of tones (Dai et al., 2018) or speech (Petersen et al., 2017) in challenging acoustic situations where hearing impairments are likely to affect sound segregation abilities. Dai et al. (2018) found less differential ERP responses to target and distractor tone streams in a spatial selective task in which HI listeners performed poorer than NH listeners. These results are consistent with the notion that cortical tracking operates on segregated “auditory objects” (Simon, 2015), whereby the inability to segregate objects (in NH and HI listeners alike) reduces the differential tracking of competing streams (Elhilali et al., 2009; Kong et al., 2015). In contrast to the present study, Petersen et al. (2017) reported a weaker differential entrainment with increasing amounts of hearing loss in a selective-listening task where HI and NH were matched in performance. This discrepancy could relate to the fact that Petersen et al. (2017) used spatially co-located speech streams at SRTs ∼80% where segregation may be challenging. To achieve similar speech performance, the level of the target speech relative to the masker speech was increased with increasing amount of hearing loss, making it difficult to isolate effects of attention from differences in target-to-masker ratios. It has thus been unclear whether listeners with hearing loss would be able to engage selective attention to suppress distracting speech in situations where the perceptual segregation of the distractor is not a bottleneck on performance.

In the present study, we addressed this by using spatially separated speech streams spoken by a male and female talker, providing both spatial cues and pitch cues for robust segregation. Despite self-reported listening difficulties, cortical synchronization to speech mixtures was found to be strongly modulated by attention in the HI listeners. Increased difficulty suppressing distractor speech in the situation where the distractor can be segregated could potentially have been associated with a less differential cortical response to the attended and ignored streams, but this was not observed. Instead, compared with age-matched NH controls, listeners with hearing loss showed (1) enhanced low-frequency envelope-entrained cortical responses, both for the attended speech (Fig. 3) and 4 Hz modulated tone stimuli (Fig. 5); and (2) a robust effect of attention on the cortical speech-entrained responses (Fig. 4). Such enhanced envelope representations could contribute to perceptual difficulties, as suggested by previous work (Millman et al., 2017). However, enhanced responses were not observed for the unattended speech. It is possible that HI listeners with altered envelope processing have to rely on attention to an even greater degree to suppress fluctuating distractor signals, and may be well trained to do so.

### Mechanisms of amplified envelope coding?

Increased envelope synchronization in the central auditory system following sensorineural hearing loss concurs with previous results (Zhong et al., 2014; Goossens et al., 2018), where the degree of enhancement has been associated with speech-in-noise deficits (Millman et al., 2017; Goossens et al., 2018). This suggests that amplified envelope coding in cortex may represent an upstream consequence of peripheral hearing damage that might itself have detrimental effects for speech-in-noise perception (Carney, 2018). It is known from speech psychophysics with NH listeners that artificially expanding the envelope of a speech signal reduces speech intelligibility (Moore and Glasberg, 1993; van Buuren et al., 1999). Envelope expansion in combination with simulated high-frequency sloping hearing loss has only minor effects on the intelligibility of a single speech signal when audibility is accounted for (e.g., by a hearing aid), but degrades the intelligibility of a speech signal in the presence of other talkers (Moore and Glasberg, 1993).

Amplified envelope coding following hearing loss has been observed previously both in the auditory periphery (Kale and Heinz, 2010) and at central stages of the auditory system (Zhong et al., 2014; Millman et al., 2017; Heeringa and van Dijk, 2019; Goossens et al., 2019). It is thus possible that effects observed in cortex could be inherited from the periphery. In presbycusis, loss of outer hair cells reduces the fast-acting compressive response of the basilar membrane in the cochlea (Ruggero and Rich, 1991). For mid- and high-level sounds, this loss of compression leads to a steeper level-response function and is considered to result in loudness recruitment (Moore and Oxenham, 1998). Kale and Heinz (2010) showed that noise-induced sensorineural hearing loss enhances the envelope synchrony of auditory nerve fiber responses, indicating that cochlear damage can lead to an enhanced envelope coding at the level of the auditory nerve.

Enhanced envelope responses observed in cortex may, however, also reflect a compensatory gain of reduced peripheral input at central auditory stages. Upregulated activity in response to cochlear damage has been observed throughout the auditory pathway (Gerken, 1979; Qiu et al., 2000; Mulders and Robertson, 2009; Sun et al., 2012; Wei, 2013). Increased excitability of central neurons could help minimize sensitivity loss, but could also lead to hyperactivity for mid- and high-level sounds (Hughes et al., 2010; Chambers et al., 2016; Salvi et al., 2017). Potential homeostatic mechanisms underlying such hyperactivity remain debated, but both cochlear damage and aging are known to decrease GABA-mediated inhibitory neurotransmission in the auditory midbrain and cortex (Caspary et al., 2008, 2013). Although decreased inhibition may help maintain mean activity levels (Turrigiano, 1999; Wang et al., 2002), it may at the same time degrade precise frequency tuning (Wang et al., 2000, 2002; Barsz et al., 2007) and accurate spike timing (Wehr and Zador, 2003; Xie, 2016), potentially affecting cues that are important for discrimination and segregation of sounds.

A number of studies have reported amplified envelope coding as an effect of aging (Walton et al., 2002; Goossens et al., 2016; Presacco et al., 2016a,b, 2019; Herrmann et al., 2017, 2019; Parthasarathy et al., 2019) also in listeners with relatively normal thresholds. Normal aging has been associated neural degeneration of auditory nerve fibers (Sergeyenko et al., 2013; Wu et al., 2019), which has also been associated with hyper-excitability in the central auditory system (Herrmann et al., 2017, 2019; Parthasarathy et al., 2019). Such cochlear neuropathy can occur without hair cell damage (Viana et al., 2015) and is likely to be further advanced in older listeners with clinical threshold shifts.

### Caveats

In our experiments, the speech stimuli were amplified to minimize effects of differences in audibility. This was done to examine potential effects of attention-driven speech processing in situations where the speech stimulus is audible. Frequency-dependent amplification based on the audiogram mirrors the situation of aided listening (e.g., with a linear hearing aid), where HI listeners experience difficulties in competing-talker situations (Fig. 2*c*). We note that matching hearing level based on the audiogram may not necessarily match the peripheral activation level and potential effects of amplification of the overall level on EEG correlates of envelope coding are undetermined.

### BCI perspectives

Finally, we note that the current results may have relevance for auditory brain-computer interfaces. In combination with speech audio separation technologies, single-trial EEG decoding of attention could be used to amplify an attended speech stream in a neuro-steered hearing instrument to help ease listening difficulties in multi-talker situations (Mirkovic et al., 2016; O'Sullivan et al., 2017). Although enhanced cortical representations of speech envelopes may not be beneficial to speech perception, they did not hinder decoding of selective auditory attention from single-trial EEG responses in older listeners with hearing loss. However, it is not clear yet how robust attention decoding would be in less favorable SNRs in HI listeners.
